# Salivary Glucose Detection with Laser Induced Graphene/AgNPs Non-Enzymatic Sensor

**DOI:** 10.3390/bios13020207

**Published:** 2023-01-30

**Authors:** Eider Pedro Aparicio-Martínez, Alejandro Vega-Rios, Velia Osuna, Rocio Berenice Dominguez

**Affiliations:** 1 Centro de Investigación en Materiales Avanzados, SC, Miguel de Cervantes #120, Complejo Industrial Chihuahua, Chihuahua 31136, Mexico; 2 CONACyT-CIMAV, SC, Miguel de Cervantes #120, Complejo Industrial Chihuahua, Chihuahua 31136, Mexico

**Keywords:** laser-induced graphene, non-enzymatic, glucose, non-invasive, silver nanoparticles

## Abstract

The tailoring of novel nanomaterials for sensitive glucose detection through a non-enzymatic mechanism is currently under intensive research. Here, we present a laser-induced graphene (LIG) electrode decorated with silver nanoparticles (AgNPs) as a catalytic element for the direct electrooxidation of glucose. The AgNPs were synthesized through cyclic voltammetry using LIG as a template, resulting in a porous tridimensional assembly with anchored nanostructures. The characterization corroborated the formation of LIG/AgNPs composite with distinctive peaks attributed to Ag_2_O and AgO interaction with glucose. The proposed non-enzymatic sensors were successfully applied for non-enzymatic amperometric detection, exhibiting a linear range from 1 to 10 mM in the first peak (+0.7 V) and a narrow range from 1 to 2 mM with higher sensitivity of 52.2 mA/mM and improved LOD of 45 μM in the second peak (+0.55 V). The applicability of the LIG/AgNPs sensor was evaluated with spiked artificial saliva in a PoC format using a smartphone potentiostat, showing an average recovery rate of 91%. The analysis was performed in a portable, mobile, and low-cost fashion using a simulated non-invasive sample, with promising results in clinical ranges.

## 1. Introduction

Glucose strips represent a significant component of the biosensing market and a primary tool for Diabetes Mellitus (DM) care worldwide. Currently, millions of patients use this enzymatic point-of-care (PoC) detector to control glycemia [1], and its demand is increasing as DM incidence rises to new levels each year [2]. Even though glucose sensors are a well-established technology, this field still requires innovative methods that exhibit accuracy, selectivity, pain-free detection using non-invasive samples, and continuous measurement [3]. For example, sensing devices with analytical capabilities to detect glucose at low concentrations in body fluids such as sweat, tears, or saliva are highly interesting [3]. Additionally, one important research topic is the design of enzyme-free devices with catalytic nanomaterials over the electrode surface to promote the direct oxidation of glucose without an immobilized biomolecule. The aim is to avoid the drawbacks of biological receptors, such as the high complexity of enzyme production, poor stability, or strict storage conditions for more favorable features such as non-temperature dependency, long-term stability, low fabrication cost, and high sensitivity [4,5,6]. Several strategies have been addressed for the design of enzyme-free glucose sensors, including composites of metallic nanoparticles (MNPs) or metallic oxide nanoparticles (MONps) with graphene. For example, 3D Cu-Co/reduced graphene oxide dendrite architectures over a pencil graphite electrode [7] and carbon/Cu composites [8], among others, have exhibited promising results. The preparation of these electrodes, in general, are by electrodeposition and pyrolysis, showing high sensitivity towards glucose. Regarding MONps-graphene composites, the most widely reported for glucose detection are ZnO, NiO, CuO, MnO, and Fe_2_O_3_ [9].

However, the current development of non-enzymatic electrochemical glucose sensors rely mainly on glassy carbon electrodes (GCE) [10,11], screen-printed electrodes (SPEs) [12,13,14], ITO [15,16] or copper tape [17]; although, novel configurations such as graphite pencil electrodes [7] and 3D carbon fiber [18] have been explored. The manufacture of low-cost and easy-to-use electrodes with novel techniques, including 3D printing and direct laser writing (DLW) can favor the design of advanced configurations with a significant impact on the final performance of the sensor [19]. Particularly, the DLW method on polyimide (PI) films with a high-power CO_2_ laser produces the formation of a porous graphene-like material known as laser induced graphene (LIG) [20,21,22,23]. In contrast to pristine graphene, LIG exhibits a tridimensional porous architecture with convenient features as electrode material, such as good conductivity, flexibility, high surface area, and abundant edge plane exposure combined with defect sites to promote high heterogeneous electron transfer [22]. Another advantage is that the DLW method is physical; thus, no toxic reagents, controlled atmosphere, and temperature monitoring are required, simplifying the synthesis and manufacture in one step. Therefore, this method allows the direct production and integration of LIG in sensing platforms, which is well suited for the mass production of low-cost disposable sensors as required in PoC systems [24].

The use of LIG electrodes combined with MNPs, e.g., Cu [25], Au@Ni [26], and Pt@Pd [27], for non-enzymatic detection have demonstrated high sensitivity and a simple manufacturing process [28]. However, LIG/silver nanoparticles (AgNPs) sensors exploiting the catalytic properties of Ag with the additional features provided by the porous 3D architecture of LIG have not yet been fully explored for direct glucose oxidation. Previously, numerous efforts for developing AgNPs/graphene composites have been presented with synthetic routes such as chemical reduction, seed mediated, electrodeposition, microwave or pulse laser ablation exhibiting attractive features such as easy fabrication, low-cost, stability, and enhanced electrochemical performance [29,30]. Considering that the action of Ag for glucose oxidation has been evaluated for non-enzymatic sensors in conventional electrode supports [17,31,32,33,34,35,36], this study explores the formation of LIG/AgNPS composites and their performance as non-enzymatic sensors for glucose. AgNPs were synthesized through cyclic voltammetry using LIG as a template, resulting in nanostructures anchored in the porous tridimensional assembly, which directly impacted detection sensitivity. Likewise, the carbon material exhibits high loading and excellent dispersion of AgNPs on the substrate. The electrode fabrication and Ag loading were optimized and characterized prior to glucose interaction analysis. Then, we evaluated the applicability of LIG/AgNPs for glucose detection in artificial saliva using a smartphone-based potentiostat. The presented approach was able to work in a POC format using a simulated non-invasive sample, with promising results in clinical ranges.

## 2. Materials and Methods

### 2.1. Materials

PI tape (127 µm) was used as a laser-induced graphene (LIG) precursor. Potassium ferricyanide (CAS no.13746-66-2), potassium ferrocyanide (CAS no.14459-95-1), glucose (CAS no. 50-99-7), dopamine (CAS no. 51-61-6), cholesterol (CAS no. 57-88-5), and lactose (CAS no. 63-42-3) were purchased from Sigma Aldrich (Toluca, Mexico). Potassium nitrate (CAS no. 7757-79-1), ascorbic acid (CAS no. 50-81-7), and fructose (CAS no. 57-48-7) were obtained from Golden Bell. Silver nitrate (CAS no. 7761-88-8) was acquired from Quimica Meyer. Potassium hydroxide (CAS no. 1310-58-3) was purchased from Fermont. During the procedures, all solutions were prepared with tridistilled water. The electrical contact for electrodes was made with silver ink from Colloidal Silver Liquid (Electron Microscopy Sciences). The Ag/AgCl ink from ALS Co Ltd (Tokyo, Japan). was used to create the reference electrode. The real sample analysis was performed using Fusuyama-Meyer artificial saliva formulation from Sigma-Aldrich. GCE (ALS Co Ltd.) and SPE DRP110 model (Metrohm, Mexico City, Mexico) were used to compare electrode performance.

### 2.2. Equipment and Material Characterization

A laser engraving machine DC-KIII CO2 (China) with a 40 W laser of 10.6 µm wavelength was used to induce graphene on the PI film. To study the material, SEM and EDS analysis were performed with a SU3600 scanning microscope (Hitachi, Tokyo, Japan). A LabRAM HR Evolution micro-Raman (Horiba, Kioto, Japan) with a green laser of 532 nm was used to characterize LIG structure. The Emstat3 blue potentiostat was used to record cyclic voltammetry (CV) using the redox couple K_3_[Fe (CN)_6_]/K_4_[Fe (CN)_6_] 5mM in KNO_3_ 1M at different sweep speeds in a potential window from −0.2 to 0.8V. The smartphone-based potentiostat Sensit Smart (PalmSens, Houten, Netherlands) was used for PoC and EIS measurements, in the latter applying a potential of 10 mV R.M.S. in a frequency range from 200 kHz to 1 Hz using the same redox couple solution. 

### 2.3. LIG Electrodes Design and Manufacture

**Figure 1:**
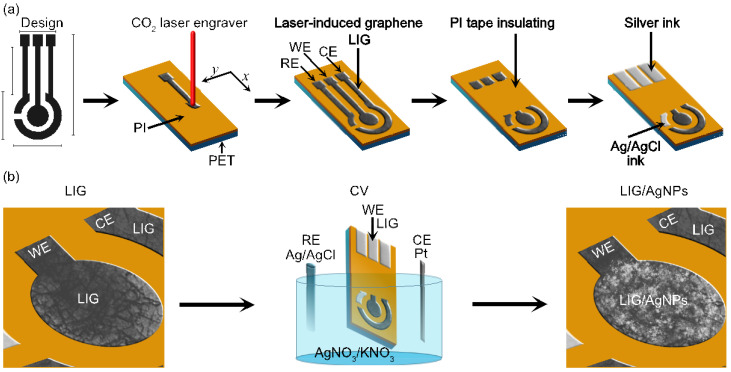
Schematic representation of LIG/AgNPs sensor fabrication: (**a**) laser-induction procedure with DC-KIII CO2; (**b**) electrodeposition of AgNPs on the working electrode (WE).

LIG fabrication was investigated through an explorative study varying laser power from 4 to 40 W and the engraving speed from 50 to 500 mm/s. Hence, LIG electrodes were manufactured under final conditions of 8W and a speed of 300 mm/s. The three-electrode configuration shown in Figure 1a was designed in CorelDraw and subsequently engraved using CorelLASER 2013.02. We use silver ink to create the electrical contact and PI tape (25 µm) to isolate the active area of the electrodes. The reference electrode (RE) was fabricated with Ag/AgCl ink, while the counter electrode (CE) was made of bare LIG.

Figure 1b illustrates the synthesis of Ag nanoparticles (AgNPs) on the working electrode (WE) of LIG. The AgNPs synthesis was carried out by electrodeposition using cyclic voltammetry with a mixture of AgNO_3_/KNO_3_ solutions. The concentrations were 0.25 mM and 1.25 mM, respectively. The potential window was from –0.8 to 0 V at a scan rate of 25, 50, and 100 mV/s.

### 2.4. Non-Enzymatic Electrochemical Glucose Detection

LIG/AgNPs sensors were investigated for glucose oxidation using a 10 mM glucose solution prepared with NaOH (0.1 M) as a supporting electrolyte and CV in a potential window from –0.2 to 1 V at 50 mV/s. Following this study, amperometry was carried out using constant potentials of 0.7 and 0.55 V with successive injections of either 1 mM or 0.2 mM, respectively.

### 2.5. Selectivity and Real Sample Evaluation

The prepared LIG/AgNPs non-enzymatic sensors were evaluated against common interferences of glucose found in physiological samples such as urea, KCl, and NaCl using the amperometric parameters for glucose detection. The real sample analysis was performed using Fusuyama-Meyer artificial saliva as a complex matrix with spike glucose concentrations.

## 3. Results and Discussion

### 3.1. Material Characterization

**Figure 2:**
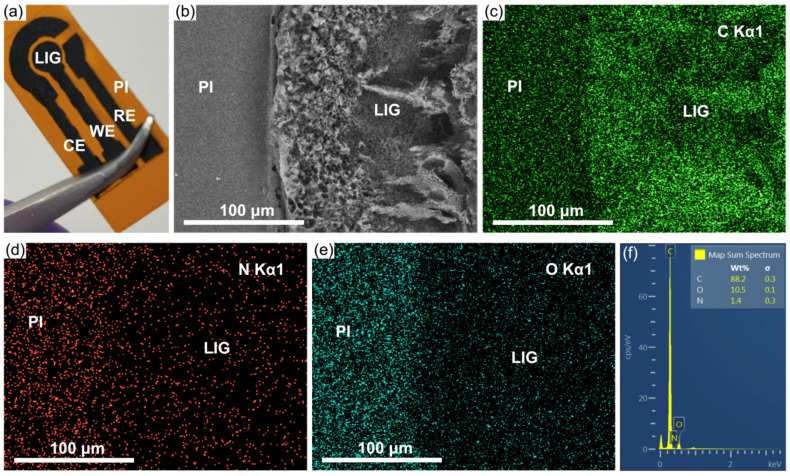
(**a**) LIG-based three-electrode system; (**b**) SEM-EDS micrography; (**c**) EDS mapping of C Kα1; (**d**) EDS mapping of N Kα1; (**e**) EDS mapping of O Kα1; (**f**) EDS elemental analysis.

Laser induction is becoming an extensively utilized technique for transducer fabrication; however, it depends on various parameters of the equipment settings, such as laser power, speed, beam focus, dpi resolution, and fluence. Thus, an exploratory analysis was performed to fabricate the LIG electrodes by varying the laser power in the DC-KIII CO2 engraving machine from 4 to 40 W and speed from 50 to 500 mm/s. Figure S1 presents the conditions evaluated (power (W) vs. speed (mm/s)) during LIG production. The areas were marked with different colors (red, yellow, blue, and green), identifying the obtained material according to the applied values. The red color indicates the burning or damage of the PI film, while the yellow color shows partial or incomplete induction. In addition, the green area depicts successful LIG formation; on the contrary, the blue color does not present any induction. Following this study, the selected final conditions for LIG electrodes were 8 W and 300 mm/s, 1000 dpi, and 5.7 cm for the Z axis. With these parameters, three-electrode systems were manufactured as presented in Figure 2a. The black region corresponds to LIG, and the orange area corresponds to the pristine PI. The LIG formation occurs due to the localized temperature increase, which produces carbonization at the polymer surface with breakage of C-O, C=O, and N-O bonds of the PI network. Figure 2b–e illustrates a SEM micrograph at the PI and LIG interface followed by a study of the surface chemical composition, particularly the elements C, N, and O. EDS analysis exhibited an increase in the carbon content in the irradiated zone compared with the pristine PI, as well as a lower presence of oxygen and nitrogen in LIG (see Figure 2c–e). The elemental analysis in Figure 2f reveals a carbon, nitrogen, and oxygen content of 88.2, 1.4, and 10.5%, respectively. Therefore, the modification of the PI surface when irradiated is confirmed.

**Figure 3:**
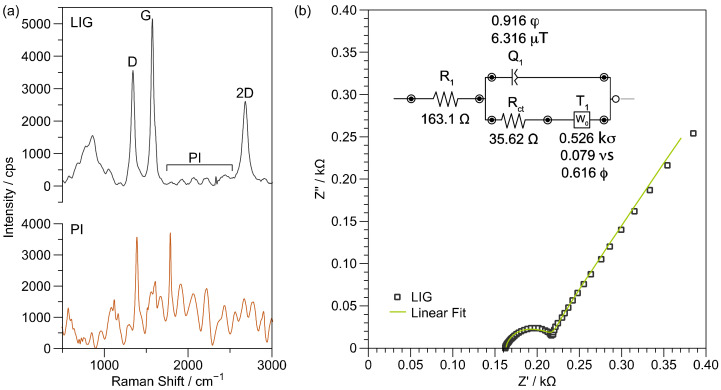
(**a**) Comparison of Raman spectra of LIG and PI; (**b**) EIS Nyquist plot of LIG-based three-electrode system.

Raman spectroscopy allows identifying the quality of carbon-based materials. In order to ensure the produced material was indeed a porous graphene structure instead of amorphous carbon, the Raman spectrum was investigated at several LIG zones and compared with PI (see Figure 3a). First, the main characteristic bands of PI are due to the imide functional group; imide I (C=O asymmetric stretch), imide II (C—N—C axial vibration), and imide III (C—N—C transverse vibration) are observed at 1788 cm^−1^, 1388 cm^−1^, and 1118 cm^−1^, respectively. Other equally important peaks are the phenylene aromatic ring that appears at 1513 cm^−1^, and the band corresponding to the aromatic amide ring in the dianhydride part at 1608 cm^−1^ [37]. Compared with the PI Raman spectrum, the formation of the D and G bands in the LIG Raman spectrum is evident. The LIG specter in Figure 3a shows the characteristic bands, including D (1342 cm^−1^), G (1575 cm^−1^), and 2D at 2681 cm^−1^. Although the D/G ratio has been frequently presented as evidence for the reduction of graphene oxide, in this case, the presence of the 2D band is unequivocally linked to the sp^2^ hybridization of the graphene electronic structure, confirming the induction of the material. In fact, complex chemical reactions have been reported to occur during PI surface irradiation. In particular, characterization using Raman spectroscopy reports the generation of D and G bands and a decrease in pristine PI peaks; although, background signals from the original PI can be present since the induction only takes place at the surface of the material [38].

Even though the Raman spectrum evidenced the structural nature of LIG layers, it is desirable to investigate the operation of LIG as an electrode. Thus, LIG was evaluated using CV and EIS with the K_3_[Fe(CN)_6_]/K_4_[Fe (CN)_6_] redox probe. First, since PI is an isolation material, no current during CV was registered, and consequently, a high impedance was obtained. However, the fabricated LIG three-electrode systems followed a quasi-reversible regimen when evaluated in CV at a speed from 5 to 50 mV/s and with an ∆E of 102 mV as observed in Figure S2. In addition, the semicircular Nyquist diagram obtained by EIS showed a calculated R_ct_ of 35.6 Ω (Figure 3b) for the fitted circuit, confirming the presence of a conductive material.

**Figure 4:**
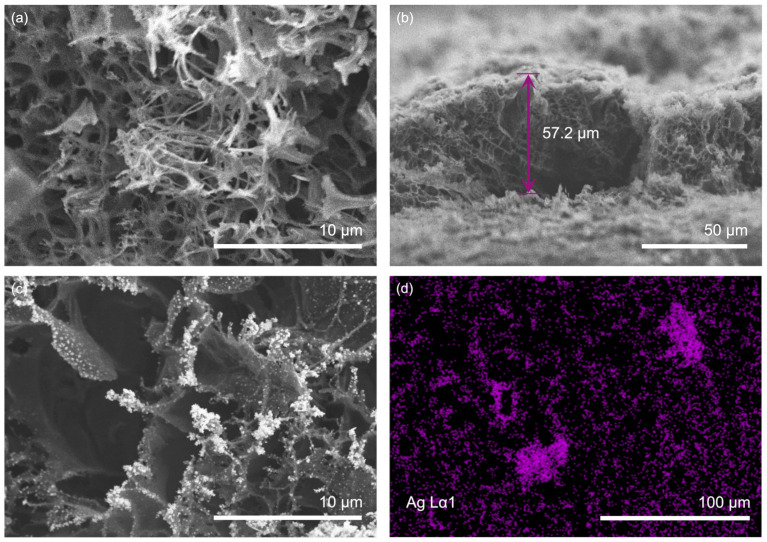
SEM micrographs: (**a**) top view of LIG; (**b**) cross-sectional view of LIG; (**c**) LIG/AgNPs; and (**d**) EDS mapping of Ag.

The changes in the morphology of the LIG and LIG/AgNPs composites were analyzed using SEM. Figure 4a illustrates the top view of the porous assembly produced after irradiation on the pristine PI surface. The morphology observed is composed mainly of intercalated fibers and exposed graphene layers at the surface. The nature of the interconnected porous structure is observed in the cross-sectional view in Figure 4b. The micrograph clearly shows the tridimensional architecture of LIG with a thickness over the pristine PI surface close to 57 μm. When LIG is used as a WE for Ag electrodeposition, the micrograph in Figure 4c shows the Ag anchoring to the graphene-exposed layers, mainly to the edge planes, which serve as nucleation sites. The use of CV for Ag growing generated smaller nanoparticles of spherical morphology, which serve as catalytic sites for glucose interaction. This CV electrodeposition also produced a homogeneous distribution of Ag over LIG electrode, resulting in higher reproducibility of measurements. Finally, the EDS of Figure 4d confirms the presence of the Ag element after electrodeposition on LIG/AgNPs electrode. Furthermore, the presence of carbon, silver, and oxygen with the contents of 85.6, 12.1, and 2.3%, respectively, are observed in elemental analysis (see Figure S3), thus confirming the formation of the LIG/AgNPs composite.

**Figure 5:**
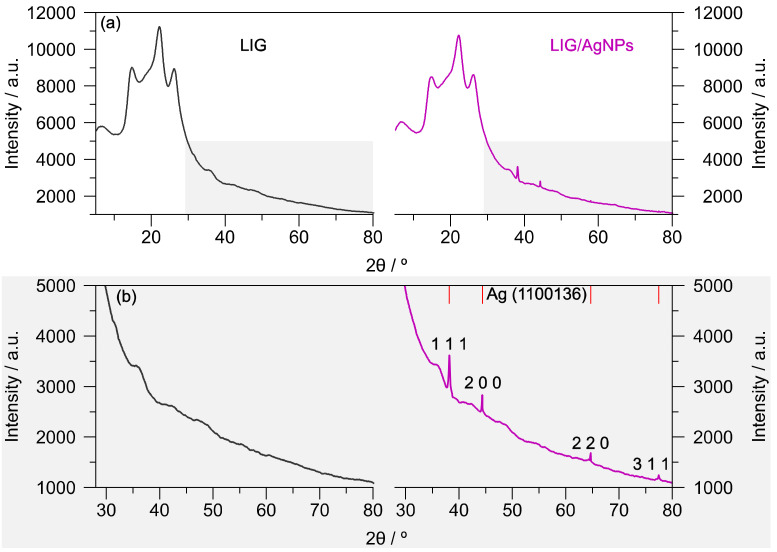
(**a**) Comparison of XRD spectra of LIG and LIG/AgNPs samples; (**b**) enlargement of the diffractogram in the region from 30 to 80°.

XRD was another technique used to study the material; the X-ray diffraction analysis of Figure 5a,b shows the peaks coming from the PI tape at 2Θ = 14.8°, 22.4°, 26.4, and 35.7°. These signals were overlapped with the LIG peaks found at 2Θ = 26° and 42.7°. Although these peaks remain in both LIG and LIG/AgNPs diffractograms, the enlargement in the region from 30 to 80° in Figure 5b shows distinctive signals in the electrodeposited material. The LIG/AgNPs diffractogram presents peaks at 2Θ = 38.2°, 44.3°, 64.5°, and 77.4°, which were indexed with the information from card 1100136 of the crystallographic open database attributed to the face-centered cubic crystal structure for AgNPs. According to the Scherrer equation, the crystallite size was determined at ~50 nm, using the peak at 2Θ = 32.8°. Similar results were observed by Uzunoglu et al. [33], utilizing a concentration of 5 mM of silver nitrate in the system of PdAg nanoparticles anchored in 3-D MWCNT-rGO nanohybrids. Likewise, Shabbir et al. [31] reported AgNPS/carbon nanotubes electrodes; however, the intensity of the peaks is higher because the concentration of the precursor of silver nanoparticles is 1 M. Moreover, the attributed signals for Ag were absent in the bare LIG diffractogram.

### 3.2. Exploratory Analysis in CV for Glucose Detection

**Figure 6:**
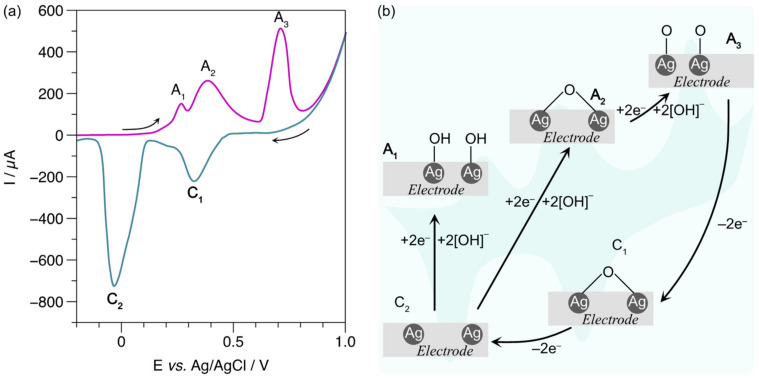
(**a**) CV of LIG/AgNPs in the presence of NaOH electrolyte; (**b**) schematic representation of Ag oxidation states in NaOH electrolyte.

Although AgNPs deposited on GCE [39,40] or ITO [41] have been presented for non-enzymatic glucose oxidation, AgNPs anchored on LIG, and their action mechanism have not been studied. The voltammograms of LIG/AgNPs with and without glucose were recorded using an alkaline solution of NaOH [0.1 M] as the supporting electrolyte. The voltammogram in Figure 6a displays three distinctive peaks in the anodic scan (magenta color) depicted as A1, A2, and A3, attributed to AgOH formation, Ag_2_O and AgO, respectively. Then, in the cathodic scan (turquoise line), the observed C1 and C2 peaks were attributed to the reduction of AgO to Ag_2_O and Ag_2_O to Ag [42,43]. In order to interpret the species produced at the electrode surface during CV, we present schematically the different oxidation states of AgNPs occurring in the absence of glucose (Figure 6b). The identification of these species is useful since the in situ produced states of A2 (Ag_2_O) and A3 (AgO) are likely to take action in the mechanism of non-enzymatic glucose oxidation, according to the model proposed by Pasta et al. [44,45].

**Figure 7:**
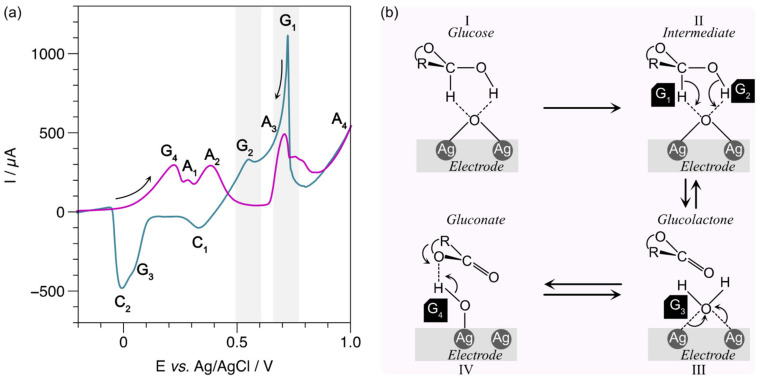
Exploratory analysis of glucose detection: (**a**) CV of LIG/AgNPs in the presence of 10 mM of glucose in NaOH; (**b**) proposed mechanism for glucose oxidation in LIG/AgNPs electrode.

Clearly, the voltammogram of Figure 7a shows new peaks in the presence of 10 mM of glucose. For example, the electroformation of Ag_2_O_3_ in the anodic sweep (magenta line) is observed at high potentials with a small anodic peak A4 [46]. The other peaks, G1, G2, G3 in the cathodic scan (turquoise line) and G4 in the anodic scan (purple line) are associated with the complex process of glucose oxidation on the LIG/AgNPs electrode. Figure 7b illustrates a proposed model to explain the new currents generated in the presence of glucose. In step 1, glucose is absorbed to the surface of the LIG/AgNPs electrode with high silver oxide content [47]. Then, the reaction of dehydrogenation of the anomeric carbon (intermediate, step II) of the glucose occurs, raising the G1 peak [44,48]. In peak G2, the alcohol proton interacts with silver oxide generating gluconolactone (step III). Afterward, the G3 peak overlaps with C2 with a peak close to a potential of 0 V [44], associated with the reduction of Ag_2_O to Ag. Finally, the complete oxidation of glucose occurs in anodic scanning at 0.2 V (G4), forming gluconate (IV). Therefore, according to the results observed in CV, the currents corresponding to G1 and G2 peaks (highlighted in gray) could be correlated with glucose concentration.

**Figure 8:**
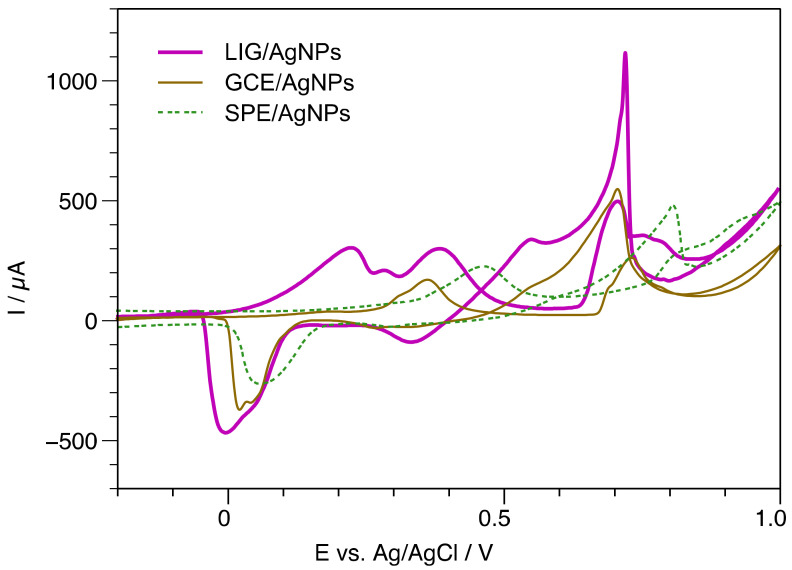
Performance comparison of LIG with SPE and GCE in the presence of glucose [10 mM].

Notably, although the sensing mechanism is driven mainly by AgNPS, the electrodeposited particle distribution, loading, and surface area provided by the unique morphological characteristics of LIG are crucial for sensitive detection when compared with other carbon electrodes, as observed in Figure 8. In this assay, GCE and SPE were used for Ag growing in the same conditions applied for LIG and evaluated in 10 mM glucose. For instance, the SPE/AgNPs electrode shows that the G1 peak shifted to 0.82V and a lower registered current. In contrast, GCE/AgNPs showed the same potential for G1 as LIG for glucose detection, but with a current of only 45% compared with that registered by the LIG/AgNPs sensor. The G2 peak is highly diminished for GCE and completely absent for the SPE substrate.

### 3.3. Amperometric Detection of Glucose

**Figure 9:**
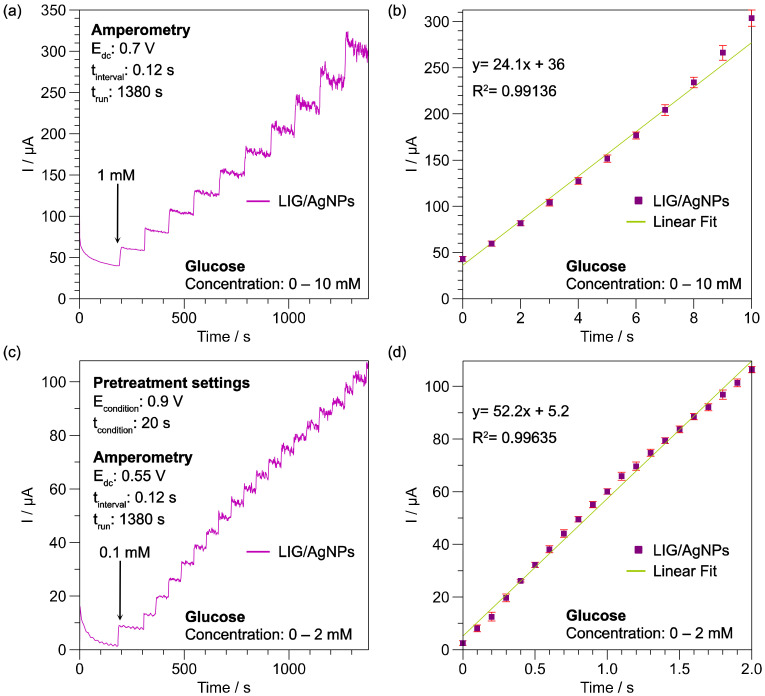
Amperometric glucose detection; (**a**) G1 region at 0.7 V and the obtained calibration curve (**b**), (**c**) G2 region at 0.55V, and the obtained calibration curve (**d**).

Based on the results of the glucose interaction observed in CV, the current generated in G1 and G2 peaks due to AgO and Ag_2_O states could be used to determine glucose concentration amperometrically using fixed potentials at 0.55 V and 0.7 V, respectively. Figure 9a shows the current vs. t curve recorded with the LIG/AgNPs-G1 sensor after glucose injection of 1 mM at fixed 0.7 V. On average, each injection produced a current of around 25 μA. Figure 9b displays the calibration curve obtained with the recorded data, exhibiting a linear range from 1 to 10 mM, a sensitivity of 24.1 μA/mM with a fitting of R^2^ = 0.99136, and a calculated limit of detection (LOD) of 412 μM. Meanwhile, the amperometric detection of LIG/AgNPs-G2 was performed at 0.55 V (see Figure 9c). A narrow detection range from 1 to 2 mM was observed; however, the calibration in Figure 9d showed a higher sensitivity of 52.2 μA/mM, a fitting of R^2^ = 0.99635, with an improved calculated LOD of 45 μM. The differences in performance were related to the Ag oxidation state interacting with glucose, which produced different current values, as observed in Section 3.2. The reproducibility of the method was calculated as 3.5% R.S.D., which is in accordance with previous reports. The selectivity of the material was evaluated after common interferences found in biological samples (e.g., serum, urine), such as urea, ascorbic acid, KCl and NaCl, in proportion to the concentration range observed in such fluids. In all cases, the response was lower than 5% of that produced by glucose, as observed in Figure S4.

To compare the capacity of the proposed LIG/AgNPs sensor with previous systems employing Ag species as a non-enzymatic catalytic material for glucose oxidation, we considered operational parameters such as working voltage, working range, and LOD (see Table 1). Notably, no approach, such as an integrated three-electrode system, was found, and mainly GCE transducers were reported. This led to incorporating other nanomaterials to an increased surface area, such as rGO or f-MWCNT. A variety of reduction methods produced the synthesis of Ag catalytic material; however, in situ production of Ag species allowed detection with both AgO and Ag_2_O in a simple manner with few steps involved.

The obtained working range and LOD are suitable for the analysis of glucose concentration in non-invasive samples, such as saliva. To evaluate the applicability of the LIG/AgNPs-G2 sensor, an assay using spiked artificial saliva as a complex matrix was prepared. In this case, the three-electrode system was evaluated in a PoC format using a smartphone potentiostat. The analysis was performed in a portable, mobile, and low-cost fashion with disposable LIG/AgNPs-G2 non-enzymatic sensors. The results showed an average recovery rate of 91% with an R.S.D. of 8.2 % for samples analyzed between 50 and 100 μM.

## 4. Conclusions

In this work, we presented a non-enzymatic glucose sensor based on an LIG electrode combined with AgNPs. The DLW technique allowed the creation of an integrated three-electrode system that was modified with AgNPs synthesized through the CV. The characterization confirmed the formation of the LIG/AgNPs composite, and the CV study showed the interaction of Ag_2_O and AgO species during glucose oxidation, highlighting the peaks G1 and G2. These configurations were explored as LIG/AgNPs-G1 and LIG/AgNPs-G2 showing a LOD of 412 and 45 μM, respectively. The analysis was performed in a portable, mobile, and low-cost fashion with disposable LIG/AgNPs as non-enzymatic sensors. The presented approach was able to work in a PoC format using a simulated non-invasive sample, with promising results in clinical ranges.

## Figures and Tables

**Table 1:** Comparison of LIG/AgNPs with Ag-based composites for non-enzymatic glucose detection.

WE	Sensing Material	Synthesis	Potential [V]	Working Range [𝛍M]	Selectivity	LOD [𝛍M]	Real Sample	Integrated System	Ref
Cu Tape	Ag	galvanic replacement	0.65	3–3300	--	1.1	serum	X	[17]
GCE	AgNPs/ f-MWCNT	chemical reduction	0.58	1.3–1000	1057.3 mA/mM	0.003	serum	X	[35]
GCE	Ag/Ag_2_O /rGO	electrodeposition	0.6	200–8000	32 μA/mM · cm	0.06	--	X	[39]
GCE	Ag/NSC/ Nafion	thermal	0.6	5–3000	--	46	--	X	[40]
LIG	AgNPs-G1	electrodeposition	0.7	0–10000	24.1 μA/mM	412	artificial saliva	YES	This work
LIG	AgNPs-G2	electrodeposition	0.55	0–2000	52.2 μA/mM	45	artificial saliva	YES	This work

## Data Availability

Not applicable.

## Supplementary Materials

The following supporting information can be downloaded at https://www.mdpi.com/article/10.3390/bios13020207/s1, Figure S1. Processing conditions of power and speed for LIG production; Figure S2. CV of LIG electrode with redox couple K_3_[Fe (CN)_6_]/K_4_[Fe (CN)_6_]; Figure S3. EDS specter of LIG/AgNPs non-enzymatic sensor; Figure S4. Interference analysis of LIG/AgNPs non-enzymatic sensor.
