# Supplementary material for: Salivary Glucose Detection with Laser Induced Graphene/AgNPs Non-Enzymatic Sensor
Source: Biosensors (Basel). 2023 Jan 30;13(2):207. doi: 10.3390/bios13020207 (PMC9954126; doi:10.3390/bios13020207)
Supplement: Supplementary file 1 [file biosensors-13-00207-s001.zip › biosensors-2090534-supplementary.pdf]

biosensors

Supplementary Information

Salivary Glucose Detection with Laser Induced Graphene/AgNPs Non-Enzymatic Sensor

Eider Pedro Aparicio-Martínez [1], Alejandro Vega-Rios [1], Velia Osuna [2] and Rocio Berenice Dominguez [2,*]

[1] Centro de Investigación en Materiales Avanzados, SC, Miguel de Cervantes #120, Complejo Industrial Chihuahua, Chihuahua 31136, Mexico
[2] CONACyT-CIMAV, SC, Miguel de Cervantes #120, Complejo Industrial Chihuahua, Chihuahua 31136, Mexico
* Correspondence: berenice.dominguez@cimav.edu.mx

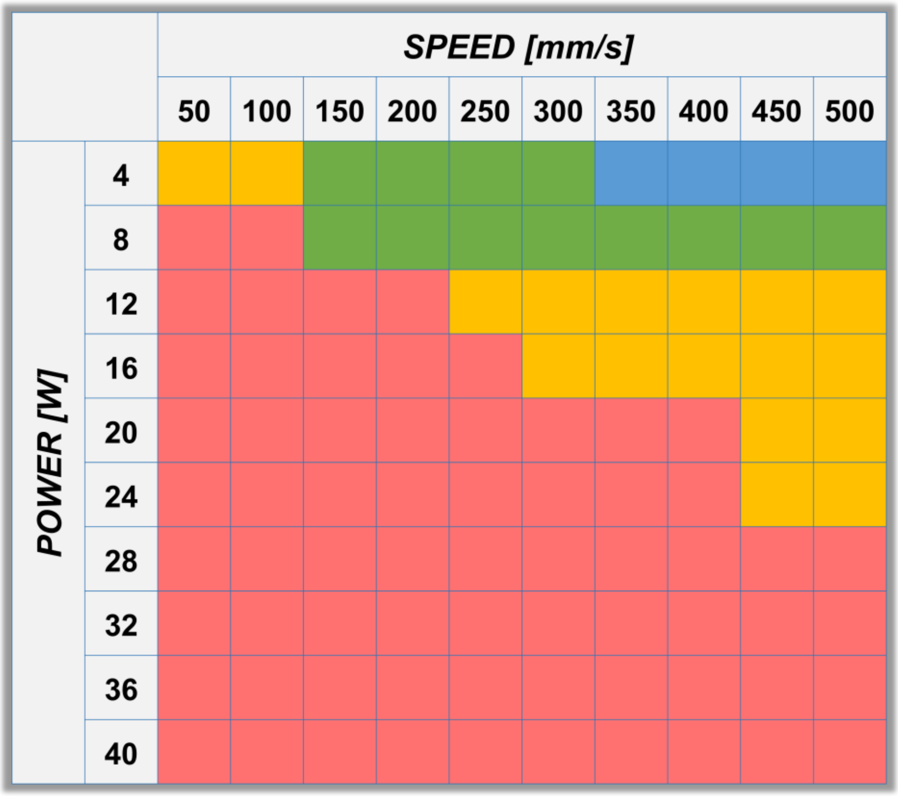

Figure S1. Processing conditions of power and speed for LIG production.

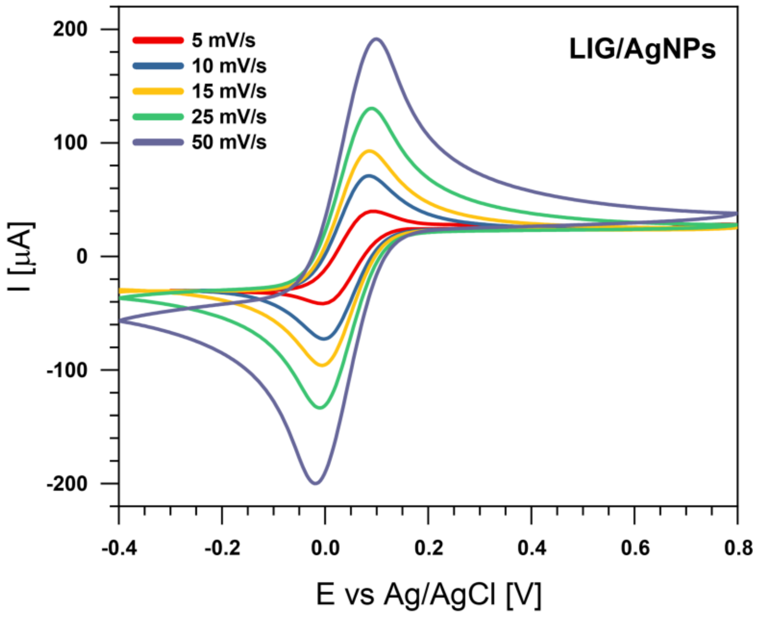

Figure S2. CV of LIG electrode with redox couple K_3[Fe $(CN)_6$]/K_4[Fe $(CN)_6$].

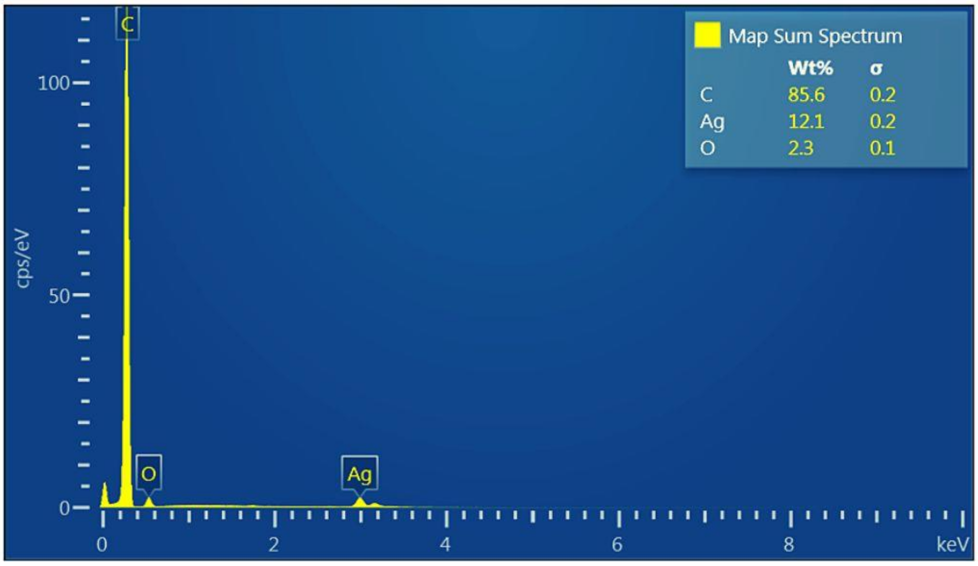

Figure S3. EDS specter of LIG/AgNPs non-enzymatic sensor.

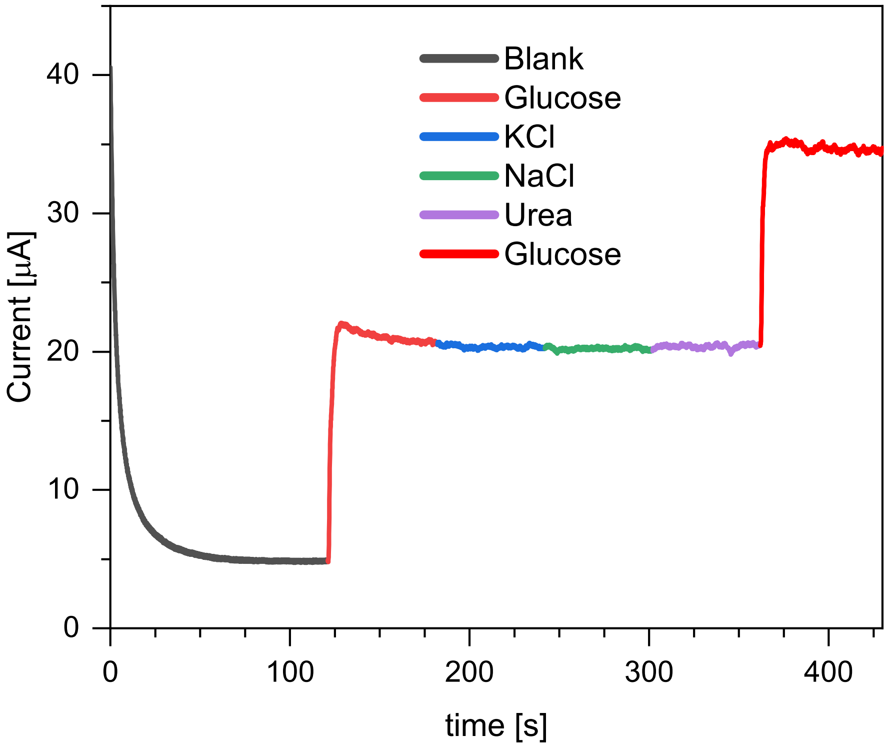

Figure S4. Interference analysis of LIG/AgNPs non-enzymatic sensor.
